# An Overview of the Practitioner Research and Collaboration Initiative (PRACI): a practice-based research network for complementary medicine

**DOI:** 10.1186/s12906-017-1609-3

**Published:** 2017-02-01

**Authors:** Amie Steel, David Sibbritt, Janet Schloss, Jon Wardle, Matthew Leach, Helene Diezel, Jon Adams

**Affiliations:** 1 Office of Research, Endeavour College of Natural Health, Fortitude Valley, QLD Australia; 2 Australian Research Centre in Complementary and Integrative Medicine, Faculty of Health, University of Technology Sydney, Ultimo, NSW Australia; 3 School of Nursing and Midwifery, University of South Australia, Adelaide, SA Australia

**Keywords:** Research, Database, Practice-based, Workforce, PBRN

## Abstract

**Background:**

The Practitioner Research and Collaboration Initiative (PRACI) is an innovative, multi-modality practice-based research network (PBRN) that represents fourteen complementary medicine (CM) professions across Australia. It is the largest known PBRN for complementary healthcare in the world and was launched in 2015. The purpose of this paper is to provide an update on the progress of the PRACI project, including a description of the characteristics of PRACI members in order to facilitate further sub-studies through the PRACI PBRN.

**Methods:**

A CM workforce survey was distributed electronically to CM practitioners across fourteen disciplines, throughout Australia. Practitioners electing to become a member of PRACI were registered on the PBRN database. The database was interrogated and the data analysed to described sociodemographic characteristics, practice characteristics, professional qualification and practice interest of PRACI members.

**Results:**

Foundational members of PRACI were found to be predominately female (76.2%) and middle-aged (82.5%). Members were primarily located in urban settings (82.5%) across the Eastern seaboard of Australia (82.5%), with few working remotely. The main modalities represented include massage therapists (58.5%), naturopaths (26.4%) and nutritionists (14.4%). The primary area of clinical interest for PRACI members were general health and well-being (75.4%), musculoskeletal complaints (72%) and pain management (62.6%).

**Conclusions:**

PRACI provides an important infrastructure for complementary healthcare research in Australia and its success relies on CM practitioners being involved in the research being conducted through the PBRN. The aim of this database is to ensure that the research conducted through PRACI is rigorous, robust, clinically relevant and reflects the diversity of clinical practice amongst CM practitioners in Australia.

## Background

The practice-based research network (PBRN) is a research infrastructure that has been growing in popularity globally since the 1960s [1, 2]. A PBRN is a group of ambulatory practices that affiliate together and collaborate with academic institutions for conducting research [3, 4]. The research conducted through a PBRN centres on data collected from patients and practitioners with an ultimate focus on improving the quality of patient care. PBRNs are commonly characterised as including at least 15 ambulatory practices and/or 15 clinicians devoted to the primary care of patients [3]. The organisational structure of a PBRN extends beyond any one single study and often encompasses administrative and managerial staff who work alongside the academic contributors and practitioner members to fulfil a shared mission and purpose in research [3].

### The complementary medicine professional landscape in Australia

The regulatory landscape in Australia for the complementary medicine professions is largely reliant on self-regulation by professional associations, with acupuncturists and Chinese herbalists being one of the few CM professions controlled through statutory registration [5]. In the absence of any national or regional databases that provide complete lists of members of the remaining CM professions, the defining features of CM professional groups rely on the broader accepted characteristics of a profession. Based on these characteristics, CM practitioners who undertake paid work which is knowledge-based and achieved following higher education and/or vocational training in their respective CM field are considered to be part of the relevant CM profession in Australia [6]. A number of practitioners may have training in multiple fields and as such may belong to multiple professions.

### The Practitioner Research and Collaboration Initiative (PRACI)

The Practitioner Research and Collaboration Initiative (PRACI) is an innovative, multi-modality PBRN that represents fourteen complementary medicine (CM) professions across Australia. The establishment and design of PRACI were reported in detail in 2014 [7] and this paper provides the first overview of members of the PRACI network. A survey of the CM practitioners that elected to join the PRACI network has since been completed. These practitioners are now founding members of PRACI and their survey responses are the baseline data of the PBRN. Each member has been allocated PRACI ID numbers that have been integrated into the PRACI database. This paper provides an update on the progress of the PRACI project and describes the characteristics of PRACI members in order to inform and facilitate further sub-studies through the PRACI PBRN.

## Methods

### Study design

A secondary analysis of the characteristics of practitioner members of the PRACI network based upon their responses to the survey used to establish the initial PRACI database.

### Aims and objectives

The aim of the paper is to describe the characteristics of members of PRACI including sociodemographic characteristics, practice characteristics, professional qualification and practice interest, to highlight opportunities for further sub-studies through the PRACI PBRN.

### Participants

All CM practitioners from targeted professions (i.e. acupuncturists, aromatherapists, Ayurvedic practitioners, Bowen therapists, Chinese herbalists, homoeopaths, kinesiologists, massage therapists, myotherapists, naturopaths, nutritionists (non-dietetic), reflexologists, Western herbalists and yoga practitioners) were invited to join the PRACI network. Completion of a baseline workforce survey was required before individual practitioners could elect to become a member of PRACI.

### The workforce survey

The PRACI database of members was established from all practitioners who completed a CM workforce survey in 2015 and elected to join the network. The questions of the baseline survey were designed to capture key information linked to each PRACI member and enable effective administration of future sub-studies through PRACI. The survey contained 19 items covering practitioner demographics and practice characteristics.

### Sociodemographic characteristics

Questions regarding the socio-demographic characteristics of participants, such as age and gender, were included. Practitioners were also asked to provide information about the number of years since completing their first qualification in complementary health care. The survey included questions about the professional qualifications of practitioners. Respondents were grouped into professional categories based on their reported qualifications (i.e. participants were only counted in the professional group of herbal medicine if they held a specific qualification in herbal medicine).

### Practice characteristics

Respondents were asked to provide information about the location of their clinical practice/s. This included the State or Territory in which they practised (or if they provided virtual clinical services), as well as the category of their practice locality (i.e. urban, rural, remote). Respondents were also asked to identify areas of practice interest in line with a wide range of common health specialities (e.g. women’s health, oncology, paediatrics, gerontology, pain management).

### Survey administration

The baseline workforce survey was distributed electronically through over 20 national professional associations, social media (e.g. Facebook, Twitter), national conferences and organisations (such as complementary health product companies and online classified lists for CM practitioners) whose client-base targeted the fourteen included professions. The survey was promoted multiple times through each organisation to maximise participation. The survey was administered between January and November 2015 using the SurveyGizmo platform.

### Analysis

Raw data was extracted from SurveyGizmo to a Microsoft Excel spreadsheet and imported into Stata 14 statistical software for analysis. Binary variables for each professional group were developed to allow for practitioners to be allocated to multiple professional groups in line with their qualifications. Similarly, binary variables were developed for the location of practice to ensure practitioners with multiple practice locations were appropriately reflected in the data. Frequencies and percentages were calculated for variables of interest for all respondents as well as for individual professional groups. In addition, associations between key demographic characteristics of PRACI members and the wider CM workforce was determined through chi-squared tests.

## Results

The baseline survey was completed by 1264 practitioners of whom 764 agreed to join the PRACI PBRN, reflecting a 60% conversion rate. The exact response rate of either the baseline survey or the PRACI membership could not be calculated due to the absence of reliable national workforce numbers, due in part to an absence of registration of the professions included in PRACI (excluding acupuncture and Chinese herbal medicine), or the age of work that has been done relating to professions in PRACI (for example, the most recent workforce data for naturopaths is from 2004 [8], which predates significant changes to the education and practice standards of that profession [9]). However, a comparative analysis of the characteristics of those who completed the survey and did not join the PRACI network, and those who did join PRACI, revealed no statistically significant differences between the two groups (see Table 1). Analysis of the characteristics of Chinese medicine practitioners registered with the Chinese Medicine Board of Australia (see Table 2) suggests a proportionally higher number of PRACI members in Queensland and South Australia and a lower number in New South Wales compared to the national distribution of total registered Chinese Medicine practitioners. Other CM modalities have conducted similar studies such as the naturopathy workforce survey, which was conducted 12 years ago [2]. As the profession has made many changes over that time, the findings from the workforce survey are valuable.

**Table 1:** Characteristics of PRACI members compared with workforce survey respondents that did not join PRACI (*n* = 1264)

	non-PRACI members (*n* = 500)		PRACI members (*n* = 764)		*p* value
Characteristics	Mean (± SE)	Confidence interval	Mean (± SE)	Confidence interval	
Age (Mean ± SE)	47.9 ± 0.5	46.9–48.9	47.8 ± 0.4	46.9–48.6	0.26
Characteristics	Frequency	Percentage	Frequency	Percentage	
Gender					
Female	393	78.6%	581	76.2%	0.26
Male	107	21.4%	182	23.9%	
State/Territory^a^					
Australian Capital Territory	7	1.4%	10	1.3%	0.76
New South Wales	65	13.0%	174	22.8%	0.14
Northern Territory	6	1.2%	4	0.5%	0.08
Queensland	96	19.2%	188	24.6%	0.83
South Australia	26	5.2%	44	5.8%	0.41
Tasmania	10	2.0%	21	2.8%	0.99
Victoria	121	24.2%	265	34.7%	0.65
Western Australia	46	9.2%	66	8.6%	0.08
Virtual	5	1.0%	10	1.3%	0.62
Location^a^					
Urban	404	80.8%	630	82.5%	0.71
Rural	120	24.0%	179	23.4%	0.56
Remote	8	1.6%	17	2.2%	0.43
Very Remote	6	0.6%	5	0.7%	0.76
Years since first qualification					
Less than 5 years	81	16.2%	147	19.2%	0.31
5 to 9 years	75	15.0%	117	15.3%	
10 to 14 years	95	19.0%	153	20%	
15 to 19 years	136	27.2%	175	22.9%	
20 years or more	113	22.6%	172	22.5%	

^a^These figures may equal more than 100% as participants may have a practice in more than one locality

**Table 2:** Characteristics of PRACI members reporting Chinese medicine qualifications compared with Chinese medicine practitioners registered with the Chinese Medicine Board of Australia (CMBA)

	AHPRA Registered Chinese Medicine practitioners (*n* = 4395)^a^		Chinese Medicine PRACI members^b^ (*n* = 100)		*p*-value
Characteristics	Frequency	Percentage	Frequency	Percentage	
Gender					
Female	2354	53.6%	62	62.0%	0.09
Male	2041	46.4%	38	38.0%	
State/Territory^c^					
Australian Capital Territory	69	1.6%	1	1.0%	0.65
New South Wales	1769	40.3%	24	24.0%	0.001
Northern Territory	17	0.4%	0	0.0%	0.53
Queensland	828	18.8%	36	36.0%	<0.001
South Australia	169	3.85%	11	11.0%	<0.001
Tasmania	35	0.8%	1	1.0%	0.82
Victoria	1235	28.0%	23	23.0%	0.26
Western Australia	229	5.0%	6	6.0%	0.73

^a^based on registrant data from December 2014 provided by the Chinese Medicine Board of Australia. This data includes 42 practitioners who are registered as a Chinese herbal dispenser only with no additional registration as an *acupuncturist* or *Chinese herbal medicine* practitioner

^b^includes practitioner members reporting to have qualifications in acupuncture and/or Chinese herbal medicine. Some PRACI members with Chinese medicine qualifications may not be registered with the Chinese Medicine Board of Australia

^c^These figures may equal more than 100% for Chinese Medicine PRACI members as participants may have a practice in more than one locality

### Sociodemographic characteristics

The sociodemographic characteristics of PRACI members have been reported in Table 1. PRACI members have a mean age of 47.8 years (min: 22 years; max: 85 years) and are predominantly female (76.2%).

### Practice characteristics

The location of PRACI members’ clinical services is presented in Table 1. Although PRACI members are represented in all Australian States and Territories, the largest number of PRACI members have a practice based in Victoria (34.7%), Queensland (24.6%) and New South Wales (22.8%), with relatively fewer practicing in Western Australia (8.6%), South Australia (5.8%), Tasmania (2.8%), Australian Capital Territory (1.3%), and Northern Territory (0.5%). A small number of members (1.3%) identify as providing a virtual clinic service in their practice. The vast majority (*n* = 630; 82.5%) of PRACI members operate a clinic based in an urban location, with a smaller proportion reporting clinical practices based in rural (*n* = 179; 23.4%), remote (*n* = 17; 2.2%) and very remote (*n* = 5; 0.7%) localities.

There is a fairly even proportional representation of PRACI members based upon the years since their first qualification in complementary health care (see Table 1). Similar frequencies are reported amongst members who first qualified 15 to 19 (*n* = 175; 22.9%) and 20 or more (*n* = 172; 22.5%) years ago, and also between members who qualified 10 to 14 years (*n* = 153; 20.0%) or less than 5 years (*n* = 147; 19.2%) ago. The least prevalent group of members based upon years since qualification are those that graduated 5 to 9 years ago (*n* = 117; 15.3%).

### Professional qualification and practice interest

The specific professional groups within which PRACI members are registered are presented in Table 2. PRACI members have strong representation in a number of professions including massage therapy (*n* = 447; 58.5%), naturopathy (*n* = 202; 26.4%), nutrition (*n* = 110; 14.4%), and reflexology (*n* = 102; 13.4%). PRACI also includes members with qualifications in acupuncture (*n* = 96; 12.6%), Western herbal medicine (*n* = 95; 12.4%), myotherapy (*n* = 69; 9.0%), aromatherapy (*n* = 60; 7.9%), homeopathy (*n* = 52; 6.8%), Chinese herbal medicine (*n* = 50; 6.5%), Bowen therapy (*n* = 49; 6.5%), kinesiology (*n* = 39; 5.1%), yoga (*n* = 30; 3.9%), and Ayurveda (*n* = 3; 0.4%).

Information about areas of special interest for practitioners was also identified for the PRACI members (see Table 3). The most common areas of special interest are general health and wellbeing (*n* = 576; 75.4%) and musculoskeletal conditions (*n* = 550; 72.0%). A substantial number of practitioners also report a special interest in pain management (*n* = 478, 62.6%), digestive disorders (*n* = 445; 58.3%), women’s health (*n* = 442; 57.9%), and mental health issues (*n* = 390; 51.1%). The least common areas of special interest are paediatrics (*n* = 251; 32.9%), renal conditions (*n* = 217; 28.4%), and gerontology (*n* = 160; 20.9%) (Table 3).

**Table 3:** Professional qualifications and practice specialities of PRACI members

Practice specialties	Frequency	Percentage
Professional qualification^a^		
Massage therapy	447	58.5%
Naturopathy	202	26.4%
Non-complementary medicine health qualification	124	16.2%
Nutrition	110	14.4%
Reflexology	102	13.4%
Acupuncture	96	12.6%
Western herbal medicine	95	12.4%
Myotherapy	69	9.0%
Aromatherapy	60	7.9%
Homeopathy	52	6.8%
Chinese herbal medicine	50	6.5%
Bowen therapy	49	6.5%
Kinesiology	39	5.1%
Yoga	30	3.9%
Ayurveda	3	0.4%
Practice specialties		
General health and wellbeing	576	75.4%
Musculoskeletal conditions	550	72.0%
Pain management	478	62.6%
Digestive disorders	445	58.3%
Women’s health	442	57.9%
Mental health	390	51.1%
Allergies and sensitivities	385	50.4%
Complex and chronic diseases	377	49.4%
Sports	373	48.8%
Endocrine health	370	48.4%
Weight	345	45.2%
Skin	332	43.5%
Cardiovascular disease	327	42.8%
Respiratory	322	42.2%
Men’s health	301	39.4%
Oncology	274	35.9%
Ear, nose and throat	269	35.2%
Paediatrics	251	32.9%
Renal	217	28.4%
Gerontology	160	20.9%

^a^These figures may equal more than 100% as participants may have a qualification in more than one profession

Details regarding the practice specialities of each profession are presented in Table 4, whilst the geographical location of PRACI members’ practices by professional group are reported in Table 5. Information from each of these tables is described below.

**Table 4:** Practice interest of PRACI members by profession

Practice specialties	Acupuncture		Aromatherapy		Ayurveda		Bowen therapy		Chinese herbal med.		Homeopathy		Kinesiology		Massage therapy		Myotherapy		Naturopathy		Nutrition		Reflexology		Western herbal medicine		Yoga	
	*n*	%	*n*	%	*N*	%	*n*	%	*n*	%	*n*	%	*n*	%	*n*	%	*n*	%	*n*	%	*n*	%	*n*	%	*n*	%	*n*	%
Allergies and sensitivities	67	69.8	41	68.3	3	100.0	20	40.8	36	72.0	43	82.7	31	79.5	179	40.0	20	29.0	139	68.8	79	71.8	58	56.9	68	71.6	14	46.7
Cardiovascular disease	63	65.6	34	56.7	3	100.0	19	38.8	33	66.0	35	67.3	19	48.7	157	35.1	22	31.9	111	55.0	64	58.2	49	48.0	59	62.1	16	53.3
Complex and chronic diseases	73	76.0	36	60.0	2	66.7	24	49.0	38	76.0	42	80.8	25	64.1	179	40.0	27	39.1	132	65.3	66	60.0	52	51.0	67	70.5	17	56.7
Digestive disorders	79	82.3	43	71.7	3	100.0	27	55.1	43	86.0	45	86.5	30	76.9	194	43.4	27	39.1	165	81.7	90	81.8	68	66.7	80	84.2	19	63.3
Ear, nose and throat	64	66.7	26	43.3	3	100.0	14	28.6	35	70.0	37	71.1	21	53.9	113	25.3	16	23.2	96	47.5	50	45.4	43	42.2	52	54.7	9	30.0
Endocrine health	65	67.7	44	73.3	2	66.7	18	36.7	33	66.0	39	75.0	25	64.1	162	36.2	18	26.0	141	69.8	76	69.1	67	65.7	73	76.8	19	63.3
Women’s health	82	85.4	47	78.3	3	100.0	25	51.0	44	88.0	45	86.5	29	74.4	205	45.9	32	46.4	160	79.2	86	78.2	69	67.6	79	83.2	23	76.7
General health and wellbeing	79	82.3	54	90.0	3	100.0	39	79.6	40	80.0	41	78.9	36	92.3	325	72.7	43	62.3	158	78.2	88	80.0	86	84.3	78	82.1	26	86.7
Gerontology	38	39.6	17	28.3	2	66.7	8	16.3	19	38.0	21	40.4	12	30.8	74	16.5	13	18.8	51	25.2	29	26.4	23	22.5	31	32.6	8	26.7
Men’s health	65	67.7	34	56.7	3	100.0	18	36.7	34	68.0	34	65.4	21	53.8	151	33.8	20	29.0	107	53.0	58	52.7	39	38.2	56	59.0	11	36.7
Mental health	69	71.9	41	68.3	3	100.0	23	46.9	37	74.0	42	80.8	29	74.4	187	41.8	24	34.8	137	67.8	78	70.9	50	49.0	72	75.8	21	70.0
Musculoskeletal conditions	83	86.5	51	85.0	3	100.0	42	85.7	42	84.0	34	65.4	32	82.0	353	79.0	65	94.2	107	53.0	60	54.6	80	78.4	57	60.0	21	70.0
Oncology	54	56.3	30	50.0	1	33.3	16	32.7	31	62.0	30	57.7	12	30.8	139	31.1	20	28.9	85	42.1	46	41.8	44	43.1	45	47.4	15	50.0
Pain management	83	86.5	46	76.7	3	100.0	31	63.3	45	90.0	32	61.5	32	82.1	282	63.1	54	78.3	103	51.0	52	47.3	67	65.7	50	52.6	16	53.3
Paediatrics	58	60.4	20	33.3	2	66.7	13	26.5	32	64.0	36	69.2	19	48.7	110	24.6	15	21.7	104	51.5	56	50.9	29	28.4	51	53.7	8	26.7
Renal	46	47.9	24	40.0	2	66.7	9	18.4	24	48.0	31	59.6	16	41.0	96	21.5	11	15.9	81	40.1	44	40.0	34	33.3	45	47.4	10	33.3
Respiratory	66	68.9	36	60.0	3	100.0	20	40.8	36	72.0	38	73.1	22	56.4	147	32.9	21	30.4	107	53.0	55	50.0	55	53.9	58	61.1	15	50.0
Skin	62	64.6	38	63.3	3	100.0	20	40.8	32	64.0	37	71.1	23	59.0	156	34.9	21	30.4	128	63.4	66	60.0	50	49.0	60	63.2	9	30.0
Sports	51	53.1	31	51.7	2	66.7	29	59.2	25	50.0	29	55.8	24	61.5	250	55.9	43	62.3	84	41.6	56	50.9	41	40.2	46	48.4	15	50.0
Weight	55	57.3	35	58.3	3	100.0	22	44.9	29	58.0	37	71.1	24	61.5	169	37.8	29	42.0	132	65.3	78	70.9	37	36.3	66	69.5	13	43.3

**Table 5:** Location of practice of PRACI members by profession

Practice specialties	Acupuncture		Aromatherapy		Ayurveda		Bowen therapy		Chinese herbal med.		Homeopathy		Kinesiology		Massage therapy		Myotherapy		Naturopathy		Nutrition		Reflexology		Western herbal medicine		Yoga	
	*n*	%	*n*	%	*n*	%	*n*	%	*n*	%	*n*	%	*n*	%	*n*	%	*n*	%	*n*	%	*n*	%	*n*	%	*n*	%	*n*	%
Australian Capital Territory	1	1.0	1	1.7	1	33.3	0	0.0	0	0.0	0	0.0	0	0.0	8	1.8	1	1.5	2	1.0	1	0.9	2	2.0	1	1.1	1	3.3
New South Wales	23	24.0	10	16.7	1	33.3	8	16.3	19	38.0	23	44.2	9	23.1	93	20.8	10	14.5	61	30.2	40	36.4	14	13.7	35	36.8	8	26.7
Northern Territory	0	0.0	0	0.0	0	0.0	0	0.0	0	0.0	0	0.0	0	0.0	1	0.2	0	0.0	1	0.5	2	1.8	0	0.0	0	0.0	1	3.3
Queensland	35	36.5	27	45.0	1	33.3	12	24.5	12	24.0	12	23.1	11	28.2	107	23.9	13	18.8	46	22.8	28	25.5	31	30.4	22	23.2	5	16.7
South Australia	10	10.4	3	5.0	0	0.0	4	8.2	4	8.0	2	3.9	1	2.6	31	6.9	3	4.4	12	5.9	4	3.6	3	2.9	10	10.5	2	6.7
Tasmania	1	1.0	0	0.0	0	0.0	5	10.2	1	2.0	0	0.0	1	2.6	15	3.4	0	0.0	3	1.5	1	0.9	5	4.9	2	2.1	1	3.3
Victoria	23	24.0	16	26.7	1	33.3	14	28.6	11	22.0	10	19.2	14	35.9	161	36.0	38	55.1	63	31.2	26	23.6	27	26.5	16	16.8	7	23.3
Western Australia	5	5.2	3	5.0	0	0.0	6	12.2	3	6.0	4	7.7	4	10.3	36	8.1	4	5.8	16	7.9	8	7.3	20	19.6	7	7.4	6	20.0
Virtual	0	0.0	0	0.0	0	0.0	0	0.0	0	0.0	1	1.9	1	2.6	1	0.2	0	0.0	5	2.5	2	1.8	0	0.0	0	0.0	0	0.0
Urban	83	86.5	49	81.7	3	100.0	37	75.5	44	88.0	44	84.6	31	79.5	364	81.4	60	87.0	161	79.7	90	81.8	85	83.3	73	76.8	25	83.3
Rural	16	16.7	13	21.7	1	33.3	13	26.5	10	20.0	11	21.2	11	28.2	108	24.2	14	20.3	49	24.3	20	18.2	24	23.5	18	19.0	6	20.0
Remote	4	4.2	3	5.0	0	0.0	1	2.0	2	4.0	1	1.9	2	5.1	7	1.6	2	2.9	4	2.0	1	1.9	2	2.0	2	2.1	1	3.3
Very Remote	1	1.0	0	0.0	0	0.0	0	0.00	1	2.0	0	0.0	0	0.0	3	0.7	0	0.0	1	0.5	1	1.9	0	0.0	0	0.0	1	3.3

#### Acupuncturists

Amongst practitioners with acupuncture qualifications, the most common areas of practice interest are pain management (*n* = 83; 86.5%), musculoskeletal conditions (*n* = 83, 86.5%) and women’s health issues (*n* = 82; 85.4%). The areas least commonly identified as practice special interests include gerontology (*n* = 38; 39.6%), renal conditions (*n* = 46; 47.9%), sports performance (*n* = 51; 53.1%), oncology (*n* = 54; 56.3%), and weight loss (*n* = 55; 57.3%). The majority of practitioners in PRACI with acupuncture qualifications are situated in urban settings (86.5%, *n* = 83) within Queensland (*n* = 35; 36.5%), New South Wales (*n* = 23; 24.0%) and Victoria (*n* = 23; 24.0%).

#### Aromatherapists

The most common areas of practice interest for aromatherapists are general health and wellbeing (*n* = 54; 90.0%) and musculoskeletal conditions (*n* = 51; 85.0%). Aromatherapists report gerontology (*n* = 17; 28.3%), paediatrics (*n* = 20; 33.3%), and ear, nose and throat conditions (*n* = 26; 43.3%) as practice areas they are least frequently interested in. Almost half of the aromatherapist members of PRACI practice in Queensland (*n* = 27; 45.0%), with a substantial proportion practicing in Victoria (*n* = 16; 26.7%) or New South Wales (*n* = 10; 16.7%). Most aromatherapists practice in an urban location (*n* = 49; 81.7%), with only one in five (21.7%, *n* = 13) based in a rural, remote or very remote setting.

#### Ayurvedic practitioners

All ayurvedic practitioners (*n* = 3) identify a focus in all areas of practice interest except complex and chronic disease (*n* = 2; 66.7%), endocrine health (*n* = 2; 66.7%), gerontology (*n* = 2; 66.7%), paediatrics (*n* = 2; 66.7%), renal conditions (*n* = 2; 66.7%), sports performance (*n* = 2; 66.7%) and oncology (*n* = 1; 33.3%). Clinical practice localities of the ayurvedic members of PRACI include Australian Capital Territory (*n* = 1), New South Wales (*n* = 1), Queensland (*n* = 1) and Victoria (*n* = 1). These practices are primarily located in urban locations (*n* = 3).

#### Bowen therapists

Musculoskeletal conditions (*n* = 42; 85.7%) and general health and wellbeing (*n* = 36; 79.6%) are most frequently identified by Bowen therapists as an area of interest in their practice. Bowen therapists are least likely to identify gerontology (*n* = 8; 16.3%), renal health (*n* = 9; 18.4%), ear, nose and throat conditions (*n* = 14; 28.6%) and oncology (*n* = 16; 32.7%) as an area of practice interest. Bowen therapist members of PRACI report practice locations in mostly urban (*n* = 37; 75.5%) settings across Victoria (*n* = 14; 28.6%), Queensland (*n* = 12; 24.5%), New South Wales (*n* = 8; 16.3%) and Western Australia (*n* = 6; 12.2%) and Tasmania (*n* = 5; 10.2%).

#### Chinese herbal medicine practitioners

Pain management (*n* = 45; 90.0%), women’s health (*n* = 44; 88.0%), digestive disorders (*n* = 43; 86.0%), and musculoskeletal conditions (*n* = 42; 84.0%) are commonly identified as an area of practice interest by practitioners with a Chinese herbal medicine qualification. Gerontology (*n* = 19; 38.0%) and renal health (*n* = 24; 48.0%) are identified as a practice interest area for Chinese herbal medicine practitioners least frequently. Practitioners with Chinese herbal medicine qualifications report practice locations in all States (most commonly New South Wales [*n* = 19; 38.0%], Queensland [*n* = 12; 24.0%] and Victoria [*n* = 11; 22.0%]) but not in the Territories. Practices for this group were primarily located in urban settings (*n* = 44; 88.0%), and to a lesser extent, rural settings (*n* = 10, 20.0%).

#### Homeopaths

A substantial number of homeopaths list women’s health (*n* = 45; 86.5%), digestive disorders (*n* = 45; 86.5%), allergies and sensitivities (*n* = 43; 82.7%), complex and chronic diseases (*n* = 42; 80.8%), and mental health (*n* = 42; 80.8%) as an area of practice interest. Gerontology (*n* = 21; 40.4%) and sports performance (*n* = 29; 55.8%) are reported least frequently as an area of practice interest by homeopaths. The practices of homeopaths are predominantly located in the States of New South Wales (*n* = 23; 44.2%), Queensland (*n* = 12; 23.1%) and Victoria (*n* = 13; 19.2%). The homeopaths in PRACI most commonly identify their practice as being located in an urban setting (*n* = 44; 84.6%), with few situated in a rural (*n* = 11; 21.2%) or remote (*n* = 1; 1.9%) location.

#### Kinesiologists

Kinesiologists report general health and wellbeing (*n* = 36; 92.3%), pain management (*n* = 32; 82.1%), and musculoskeletal conditions (*n* = 32; 82.0%) most commonly as areas of practice interest. Least common areas of practice interest amongst kinesiologists are oncology (*n* = 12; 30.8%), gerontology (*n* = 12; 30.8%), and renal health (*n* = 16; 41.0%). Kinesiologists most commonly report practice locations in Victoria (*n* = 14; 35.9%) although a substantial number practice in Queensland (*n* = 11; 28.2%), and New South Wales (*n* = 9; 23.1%). Practices in Western Australia (*n* = 4; 10.3%), South Australia (*n* = 1; 2.6%), and Tasmania (*n* = 1; 2.6%) are less common. The majority of kinesiologists’ practice locations are reported in the urban setting (*n* = 31; 79.5%), followed by the rural setting (*n* = 11; 28.2%).

#### Massage therapists

The most commonly identified areas of practice interest for massage therapists are musculoskeletal conditions (*n* = 353; 79.0%), general health and wellbeing (*n* = 325; 72.7%), and pain management (*n* = 282; 63.1%). Gerontology (*n* = 74; 16.3%), renal health (*n* = 96; 21.5%), and paediatrics (*n* = 110; 24.6%) are least commonly reported. While all States and Territories have massage therapists as members of PRACI, the majority of massage therapists in PRACI have a practice location in Victoria (*n* = 161; 36.0%), Queensland (*n* = 107; 23.9%), and New South Wales (*n* = 93; 20.8%). The massage therapists practice primarily in an urban location (*n* = 364; 81.4%), and to a lesser extent, in a rural location (*n* = 108; 24.2%).

#### Myotherapists

The areas of particular practice interest for myotherapists are musculoskeletal conditions (*n* = 65; 94.2%) and pain management (*n* = 54; 78.3%). Least common areas of practice interest amongst the PRACI myotherapists are renal health (*n* = 11; 15.9%), gerontology (*n* = 13; 18.8%), and paediatrics (*n* = 15; 21.7%). More than half of the myotherapists have their practice located in Victoria (*n* = 38; 55.1%) although a number are also situated in Queensland (*n* = 13; 18.8%) and New South Wales (*n* = 10; 14.5%). Whilst the majority of myotherapist members of PRACI practice in an urban location (*n* = 60; 87.0%), one in five also report practising in a rural setting (*n* = 14; 20.3%).

#### Naturopaths

Naturopaths most commonly report digestive disorders (*n* = 165; 81.7%), women’s health (*n* = 160; 79.2%) and general health and wellbeing (*n* = 158; 78.2%) as areas of practice interest. Least common is gerontology (*n* = 51; 25.2%), renal health (*n* = 81; 40.1%) and sports performance (*n* = 84; 41.6%). A similar number of naturopaths who are members of PRACI practice in Victoria (*n* = 63; 31.2) and New South Wales (*n* = 61; 30.2%), with slightly lower numbers reported in Queensland (*n* = 46; 22.8%) and less again in other States and Territories. The PRACI naturopaths report practicing mostly in urban (*n* = 161; 79.7%) and rural (*n* = 49; 24.3%) settings.

#### Nutritionists

The most frequently cited areas of practice interest for nutritionists are digestive disorders (*n* = 90; 81.8%), general health and wellbeing (*n* = 88; 88.0%) and women’s health (*n* = 86; 78.2%). Gerontology (*n* = 29; 26.4%), renal health (*n* = 44; 40.0%) and oncology (*n* = 46; 41.8%) are reported least frequently by nutritionists as areas of practice interest. The highest number of nutritionists who are members of PRACI are located in New South Wales (*n* = 46; 36.4%), Queensland (*n* = 28; 25.5%) and Victoria (*n* = 26; 23.6%), with PRACI nutritionists primarily practicing in urban (*n* = 90; 81.8%) and rural locations (*n* = 20; 18.2%).

#### Reflexologists

General health and wellbeing (*n* = 86; 84.3%), musculoskeletal conditions (*n* = 80; 78.4%), and women’s health (*n* = 69; 67.6%) are listed as areas of practice interest amongst the majority of reflexologists, whereas gerontology (*n* = 23; 22.5%), paediatrics (*n* = 29; 28.4%) and renal health (*n* = 34; 33.3%) are listed as areas of practice interest by smaller numbers of PRACI reflexologists. The practice locations of reflexologists involved in the PRACI network are reported in all States and Territories except Northern Territory, with the highest number occurring in Queensland (*n* = 31; 30.4%), Victoria (*n* = 27; 26.5%), and Western Australia (*n* = 20; 19.6%). The majority of PRACI reflexologists describe their practice setting as urban (*n* = 85; 83.3%) or rural (*n* = 24; 23.5%).

#### Western herbal medicine practitioners

Digestive disorders (*n* = 80; 84.2%), women’s health (*n* = 79; 83.2%), and general health and wellbeing (*n* = 78; 82.1%) are all practice interest areas commonly identified by PRACI Western herbal medicine practitioners. In contrast, gerontology (*n* = 31; 32.6%), oncology (*n* = 45; 47.4%), renal health (*n* = 45; 47.4%) and sports (*n* = 46; 48.4%) are least commonly identified as areas of practice interest amongst the PRACI Western herbal medicine practitioners. Over half of Western herbal medicine practitioners list their practice location as either New South Wales (*n* = 35; 36.8%) or Queensland (*n* = 22; 23.2%), although a substantial number are located in Victoria (*n* = 16; 16.8%), South Australia (*n* = 10; 10.5%) and Western Australia (*n* = 7; 7.4%). Most Western herbal medicine practitioners describe their practice as being located in an urban setting (*n* = 73; 76.8%), with only 19% (*n* = 18) located rurally.

#### Yoga practitioners

Yoga practitioners who are members of PRACI most frequently report general health and wellbeing (*n* = 26; 86.7%), women’s health (*n* = 23; 76.7%), mental health (*n* = 21; 70.0%) and musculoskeletal conditions (*n* = 21; 70.0%) as areas of practice interest. Gerontology (*n* = 8; 26.7%) and paediatrics (*n* = 8; 26.7%) are least frequently reported by yoga practitioners as an area of practice interest. Yoga practitioners are located in all States and Territories, although the highest numbers are in New South Wales (*n* = 8; 26.7%), Victoria (*n* = 7; 23.3%), Western Australia (*n* = 6; 20.0%), and Queensland (*n* = 5; 16.7%). These practitioners describe their practices as mostly situated across urban (*n* = 25; 83.3%) and rural (*n* = 6; 20.0%) settings.

## Discussion

This paper presents an overview of the characteristics of the practitioners who are the foundational members of the PRACI network. The findings indicate a membership that is predominantly female, and middle-aged, which is consistent with the profiles described in previous Australian complementary health care workforce research [10]. This suggests that the PRACI workforce may closely approximate the national CM workforce, although this is an area warranting further exploration. The findings of the survey also point to a membership that is primarily located in urban settings across the Eastern seaboard of Australia, with very few working in remote/very remote locations or Australian territories. This almost certainly reflects the population distribution in Australia, and accordingly, the areas where there is likely to be much higher demand for such services.

### Future directions

#### Follow-up surveys

The next stage of the PRACI project will be to draw upon and expand the foundational PRACI database reported here via a series of follow-up surveys [7]. These follow-up surveys will provide more detailed information about the practice characteristics of PRACI members and will consist of four interrelated yet discrete surveys, each targeting one of four practitioner categories: the Manual Therapies survey for massage therapists, myotherapists, Bowen therapists, reflexologists and aromatherapists; the Traditional Chinese medicine survey for acupuncturists and Chinese herbal medicine practitioners; the Ingestive Medicines survey for homeopaths, naturopaths, Western herbal medicine practitioners and nutritionists; and the Yoga survey for yoga practitioners. These follow-up surveys will examine additional aspects of clinical practice including details of prescribing practice, patient populations, use and attitudes to research, and other practice relevant details. All survey responses will be linked back to PRACI member profiles through the use of PRACI ID numbers [7]. Once completed, it is expected that these surveys will provide additional data to support the conduct of targeted sub-studies through the PRACI network. Future analyses will be published which integrates the results from the baseline survey and follow-up surveys for each profession.

#### Sub-studies

Sub-studies are a cornerstone to the ongoing success and contribution of PRACI. Unlike practice-based research networks which draw upon ‘big data’ [11], PRACI utilises a sub-study structure that facilitates a diverse range of research questions and designs. In the case of PRACI, this diversity is substantial due to the breadth of professions within the network and the ability to target practitioners with specific areas of interest or who practice in different geographical locations. In line with the common characteristics of PBRNs [3], PRACI has been established independent of any single study and the PRACI network will be accessible to national and international researchers seeking to undertake research within a CM clinical setting. Whilst not intended to be exhaustive, some examples of the types of research design that may be employed through PRACI substudies are outlined below.

##### Clinical trials

Clinical trials require both a clinician and a clinical environment in order to successfully implement the intervention. Ideally, for the findings to be as clinically relevant as possible, the trial should also be conducted in a ‘real life’ setting [12]. PRACI provides the opportunity for researchers to access both clinics and clinicians for the conduct of clinical trials. This opportunity allows not only for single-centre but also multi-centre clinical trials conducted by qualified researchers. Multi-centre clinical trials (i.e. any clinical trial where there is more than one site through which the study is conducted) are well-regarded as they provide additional rigour to the clinical trial study design. Multi-centre clinical trials strengthen the external validity of research findings, provide more sensitive information regarding effect size, and may compensate for issues regarding clinician blinding [13]. It is also recommended that clinicians delay integrating new practices into their clinical decision-making until there is evidence drawn from multi-centre clinical trials to support the practice [13, 14]. For this reason, CM practitioners and researchers interested in implementing evidence-based practice into clinical settings will benefit substantially from multi-centre clinical trials conducted through PRACI. Researchers will also gain assistance from the PRACI network in refining the research question and intervention before conducting a trial with PRACI members providing a panel of experts—a feature that has been recommended to help ensure quality multi-centred trials [14]. Furthermore, based upon the number of sites needed for a multi-centre clinical trial, all PRACI professional groups would potentially be able to support a study of this type.

##### Observational studies

Retrospective, prospective or cross-sectional observational studies targeting patients of CM practitioners or the practitioners themselves, can be conducted through PRACI. Due to the scale and breadth of PRACI, the volume of patients who can be accessed through the PRACI network is substantial and potentially allows large samples to be recruited over a short timeframe. PRACI can also facilitate the administration of surveys to a small number of patients across a large number of practitioners, thereby reaching a statistically powerful sample with relative ease. The ability to implement surveys across multiple settings also minimises sampling bias and adds to the rigour of the findings [15].

Across a number of the professional groups represented in the PRACI network, there is also the capacity to undertake surveys of the practitioners themselves to better understand aspects of their practice and clinical experience in more detail. Findings from such studies can be used, for example, to support the development of interventions for clinical trials or simply to generate a better understanding of daily routine practice approaches amongst grass-roots clinicians [16].

##### Qualitative projects

PRACI also has the flexibility to accommodate qualitative research projects ranging from semi-structured interviews through to non-participant observation of practitioner-patient interactions. Such projects can constitute standalone projects or be one part of a mixed method research designs. The employment of qualitative research designs through PRACI has the capacity to support a deeper understanding of the meaning underpinning CM practitioners’ and patients’ experiences, attitudes and behaviours [17]. As qualitative research is focused more on understanding the richness rather than the breadth and generalisability [17], research projects employing qualitative methods would be supported through all practitioner groups in PRACI.

##### Case series

PRACI has the capacity to support the collection of case studies and the development of case series publications within CM. Data for a case series can be collected retrospectively through practitioners providing summarised information of existing case records using data extraction tables provided by a research team [18]. They can also be undertaken as prospective case series through which a researcher can work with practitioners identified as having a special interest in the management of a particular condition. Such an approach could be used to develop agreed outcome measurement tools (such as validated patient-reported outcome measures) and/or plan ongoing data collection for publication [18].

#### Dissemination of findings

The PRACI Steering Committee considers the dissemination of findings of PRACI research projects and sub-studies to be of the utmost importance. A newsletter to PRACI members will be sent out quarterly which will include updates on sub-studies to ensure participants are aware of the progress of any project they have been involved in. The findings from the baseline workforce survey used in the formation of PRACI are also being written up and submitted to peer-reviewed journals. A report will also be produced from this workforce data and made publicly available on the PRACI website. Dissemination will also occur through research and practitioner conferences, and national and electronic print media.

To support this work, a policy and procedure document for data access, analysis and publication of PRACI research has been developed by the PRACI Steering Committee and relates to all outcomes of research undertaken through PRACI. Requirements for the dissemination of findings outlined in this document include appropriate attribution of authorship, compliance with institutional and National Health and Medical Research Council guidelines, and acknowledgement of PRACI, Endeavour College of Natural Health and PRACI members in all publications. It is also a requirement of research teams undertaking sub-studies through PRACI that they provide copies of manuscripts and published papers to the PRACI Steering Committee.

#### Recruitment and database maintenance plans

The PRACI network is not intended to be a static resource. The PBRN needs to be flexible as the practitioner populations change over time. This includes the ability to accommodate for new graduates entering the various professions, and for the attrition of existing PRACI members from their profession. For this reason, the PRACI network will be open to new members in the future, at which time existing members will be contacted to confirm their continuation as a clinician in their profession. This process will assist the PRACI team to maintain the currency and relevance of the PRACI network.

## Conclusions

PRACI is a new and innovative resource for CM research. It represents the largest CM practice-based research network in the world based on the total number of included professional groups (*n* = 14). It also has a high level of representativeness based on existing workforce data and has the capacity to support a diverse range of research designs as sub-studies. For this reason, PRACI is expected to make a significant contribution to CM research in Australia and throughout the world. Our aim is to ensure that the research conducted through PRACI is rigorous, robust, clinically relevant and reflects the diversity of clinical practice amongst CM practitioners in Australia.

## Abbreviations

CM: Complementary medicine
HREC: Human Research Ethics Committee
PBRN: Practice-based research network
PRACI: Practitioner Research and Collaboration Initiative
